# Small supernumerary marker chromosomes (sSMC) in humans; are there B chromosomes hidden among them

**DOI:** 10.1186/1755-8166-1-12

**Published:** 2008-06-04

**Authors:** Thomas Liehr, Kristin Mrasek, Nadezda Kosyakova, Caroline Mackie Ogilvie, Joris Vermeesch, Vladimir Trifonov, Nikolai Rubtsov

**Affiliations:** 1 Institute of Human Genetics and Anthropology, Kollegiengasse 10, D-07743 Jena, Germany; 2 Research Centre for Medical Genetics, Russian Academy of Medical Sciences, Moskvorechie Str. 1, Moscow 115478, Russian Federation; 3 Division of Medical and Molecular Genetics, King's, Guy's and St. Thomas' Medical School, London, UK; 4 Center for Human Genetics, University Hospital Leuven, Herestraat 49, B-3000 Leuven, Belgium; 5 Department of Clinical Veterinary Medicine, Madingley Road, CB3 OES Cambridge, UK; 6 Institute of Cytology and Genetics, Lavrentev Str. 10, 630090 Novosibirsk, Russia

## Abstract

**Background:**

Small supernumerary marker chromosomes (sSMC) and B-chromosomes represent a heterogeneous collection of chromosomes added to the typical karyotype, and which are both small in size. They may consist of heterochromatic and/or euchromatic material. Also a predominance of maternal transmission was reported for both groups. Even though sSMC and B-chromosomes show some similarity it is still an open question if B-chromosomes are present among the heterogeneous group of sSMC. According to current theories, sSMC would need drive, drift or beneficial effects to increase in frequency in order to become B chromosome. However, up to now no B-chromosomes were described in human.

**Results:**

Here we provide first evidence and discuss, that among sSMC B-chromosomes might be hidden. We present two potential candidates which may already be, or may in future evolve into B chromosomes in human: (i) sSMC cases where the marker is stainable only by DNA derived from itself; and (ii) acrocentric-derived inverted duplication sSMC without associated clinical phenotype. Here we report on the second sSMC stainable exclusively by its own DNA and show that for acrocentric derived sSMC 3.9× more are familial cases than reported for other sSMC.

**Conclusion:**

The majority of sSMC are not to be considered as B-chromosomes. Nonetheless, a minority of sSMC show similarities to B-chromosomes. Further studies are necessary to come to final conclusions for that problem.

## Background

### Small supernumerary marker chromosomes (sSMC)

Small supernumerary marker chromosomes (sSMC) are a major clinical problem, especially when detected prenatally during banding cytogenetic analysis. sSMC have been defined as structurally abnormal chromosomes that cannot be identified or characterized unambiguously by conventional banding cytogenetics alone, and are (in general) equal in size or smaller than a chromosome 20 of the same metaphase spread. As they are too small to be considered for their chromosomal origin by traditional banding techniques; molecular cytogenetic techniques (including array based comparative genomic hybridization) are needed for their characterization [1]. The risk for an abnormal phenotype in prenatally ascertained *de novo *cases with sSMC is given as ~13%. This has been refined to 7% (for sSMC from chromosome 13, 14, 21 or 22) and 28% (for all non-acrocentric autosomes) [2] and recently been suggested to be 30% for all sSMC carriers [3]. Lately familial sSMC turned out to be transmitted predominantly via the maternal line [4]. With a newborn rate of 0.044% for all sSMC there are presently ~2.7 × 10^6 ^carriers of sSMC worldwide [3]. However, still the statement of Paoloni-Giacobino et al. (1998) [5] is valid, i.e. that cases with a *de novo *sSMC are not easy to correlate with a clinical outcome, even though first approaches in that direction where recently done [6]. With respect to current technical developments in molecular cytogenetics, i.e. fluorescence in situ hybridization (FISH), like cenM-FISH techniques [7] and molecular genetic approaches as array-CGH (e.g. [8]) further progress in this field is to be expected. These advances are clinically important as in a certain percentage of potentially healthy children with sSMC still an unnecessary abortion is induced [3]. sSMC are scientifically interesting due to their still not completely understood mode of formation, karyotypic evolution and the fact, that their presence may lead to chromosomal imbalances (partial tri-, tetra- or hexasomies) without detectable clinical consequences [1,3,6,9,10].

### B-chromosomes

B chromosomes are "additional passengers found in the karyotypes of about 15% of eukaryote species. They are best understood as genome parasites exploiting the host genome because of their transmissional advantage, and are frequently not deleterious for the organism carrying them". B chromosomes were described for plants, fungi, insects, helminth parasites, crustaceans, fish, amphibians, reptiles, birds and mammals [11]. The evolution of B chromosome mainly depends on two properties: drive (= transmission rate) and effects on fitness. For old B chromosome systems, it is conceivable that they might have evolved towards neutrality (no drive or fitness effects), but it is unlikely that young extra chromosomes lacking drive or beneficial effects (even being neutral) might invade a population and become B chromosomes [12].

### sSMC and B-chromosomes

There are several similarities between sSMC and B-chromosomes: they represent a heterogeneous collection of chromosomes added to the standard karyotype, they are small in size, may consist of heterochromatic and/or euchromatic material, there is predominance of maternal transmission and they demonstrate a tendency to mitotic instability [1,3-5,11-13]. Most human sSMC seem to be evolutionary young elements, as still their origin may be tracked to another human chromosome through molecular analyses. Thus, according to current theories, sSMC would need drive, drift or beneficial effects to increase in frequency in order to become B chromosomes [10-13]. Yet, no B- chromosomes were detected in the human species.

Here we hypothesize and provide first evidence that among the sSMC B-chromosomes might be hidden. If so, this would have also impact on the clinical interpretation of familial sSMC.

The question as to whether sSMC can be interpreted as in some way equivalent to the B chromosomes reported in other species has been the subject of debate and discussion in the literature. However, in summary, it is thought that most sSMC are not B chromosomes [13]. Nonetheless, there are at least two potential candidates which may already be, or may in future evolve into, B chromosomes: (A) sSMC stainable only by DNA derived from itself [14] and (B) acrocentric-derived inverted duplication sSMC without associated clinical phenotype [1,3,6,10].

How can sSMC gain a evolutionary significant drive? (1) either by a high transmission rate or (2) by a recurrent origin – both would be equivalent to increase sSMC frequency in the population. It seems unlikely that sSMC presence could be (3) beneficial for carrier fitness and a drive could function like that; however, with some help from drive or genetic drift, a neutral sSMC might spread in a population (i.e. neutral on fitness and drive or drift) [12]. (4) Another condition for an sSMC to develop via B chromosome is that the sSMC should reach a polymorphic status, i.e. the same sSMC (with roughly the same molecular nature) should be present in a number of non-relative individuals of the same population. (5) An sSMC should possibly show some differences in molecular nature in respect to A chromosomes.

## Results and discussion

### (A) sSMC stainable only by DNA derived from itself

**Figure 1:**
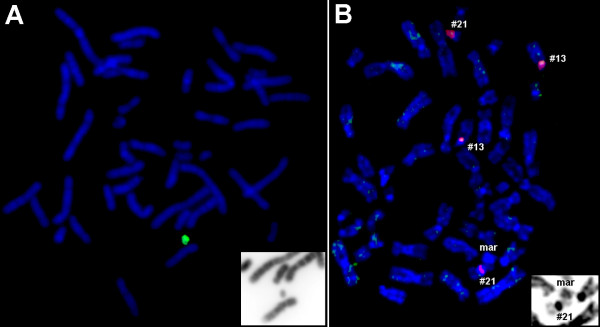
**A) Result of microdissection and reverse painting of the here reported new case with an sSMC stainable only by DNA derived from itself.** In the right lower edge: inverted DAPI for the sSMC. B) Reverse painting with the microdissection derived probe shown in Fig. 2A to the case with an sSMC stainable only by DNA derived from itself [14]. No specific green FISH-signal was obtained here; as a positive control for FISH a centromeric probe for chromosome 13/21 (Q-BIOgene) was applied in parallel (red signal). In the right lower edge: inverted DAPI for the sSMC.

Up to now there was only one case described in which a DNA probe derived from the flow sorted sSMC painted only the sSMC itself and no signal was detected on any other chromosomal regions [14]. Here we report on a second case where similar results were obtained: a DNA probe obtained from the corresponding sSMC by glass-needle based microdissection [8] did not stain any other chromosomal region than the sSMC itself in reverse FISH (see Fig. 1a). Moreover, the here reported sSMC was not stained by any centromeric probe using centromere-specific multicolor-FISH (cenM-FISH) [7], or multicolor-FISH applying all human whole chromosome painting probes (M-FISH) [15]. However, a probe specific for human Alu-repeat sequences [16] painted the sSMC as well as all other chromosomes, thus providing evidence for the origin of the sSMC from some part of the genome (result not shown). Probably the region of origin is too small to be detected by FISH experiments. We also performed FISH using the sSMC-DNA of our case as probe on metaphase spreads of the previously reported one [14], however, the DNA of both cases seemed not to be identical, as no FISH signals were obtained on that sSMC (Fig. 1b). Interestingly, our case was clinically normal, whereas that of Mackie Ogilvie et al. (2001) [14] showed some dysmorphism and mild developmental delay. The sSMC described in [14] was also studied by a whole genomic array-CGH analysis and this did not reveal any specific results (data not shown). These observations may tentatively suggest the presence of a small human population with B chromosome-like sSMC, with no, or only very mild, clinical phenotype. However, inheritance of this kind of sSMC has not yet been demonstrated.

### (B) acrocentric-derived inverted duplication sSMC without associated clinical phenotype

It is well established that about two thirds of reported sSMC are derived from acrocentric chromosomes. According to [10] almost 70% of those acrocentric derived sSMC are inverted duplicated, dicentric derivatives, which do not carry any euchromatin, and may be transmitted throughout several generations. For the most frequent subgroup, sSMC derived from chromosome 15, ~50% of carriers are clinically normal [17]. According to the newborn rate of 0.044% for and the data provided in [1,3] for sSMC(15) without clinical effect, at least 375,000 people are presently carriers of this kind of sSMC. This may be an underestimate, as most sSMC are detected as incidental findings in healthy people studied cytogenetically, mainly due to fertility problems. Such sSMC(15) behave in a similar way to B chromosomes – there have even been cases described carrying two such sSMC(15), with no phenotypic effects ([18-20] case 6, [21] case 1).

However, in the available data on sSMC collected on the sSMC homepage (2007) there is always a bias, as mainly 'interesting' sSMC-cases are reported throughout the literature and sSMC which are not correlated with clinical problems are not as likely to be described as sSMC in connection with clinical abnormalities. This is also relevant for the data included in Table 1, where the parental and chromosomal origin of all sSMC cases reported in detail is listed separately for non-acrocentric = sSMC(n-acro) and acrocentric derived sSMC = sSMC(acro). In summary, only 168/1488 (= 11.3%) sSMC cases collected in Tab. 1 are familial cases, while it is known from population studies that 30% of all sSMC are familial [3]. However, when comparing inheritance of non-acrocentric to acrocentric chromosome derived sSMC there is no reason to suggest a similar bias for this data. And here we find that in sSMC(acro) 3.9× more familial cases are reported than in sSMC(n-acro). This can mean several things. (i) There is *per se *a higher frequency of viable, healthy individuals with sSMC(acro) versus such with sSMC(n-acro); (ii) sSMC(acro) tend to be more stable in their inheritance throughout the generations than sSMC(n-acro); (iii) there is a subset of the sSMC(acro) already behaving in a similar way to B-chromosomes and spreading in the population.

**Table 1:** Parental and chromosomal origin of all in detail reported sSMC cases, summarized according [10].

Chromosomal origin	inherited	*de novo*	unclear	in summary
Non-acrocentric chromosomes				

1	1	41	9	51
1/5/19	0	5	1	6
2	2	15	10	27
3	3	12	15	30
4	0	15	5	20
5	1	16	9	26
6	2	12	6	20
7	2	16	6	24
8	2	59	20	81
9	5	48	10	63
10	0	12	4	16
11	1	7	3	11
12	3	187	5	195
16	3	24	11	38
17	0	19	5	24
18	3	160	10	173
19	1	23	3	27
20	2	22	7	31
X	1	19	10	30
Y	2	8	3	13
Summary	34	720	152	906 *754 cases with known parental origin*

i.e. 34/754 of all reported detailed characterized n-acro sSMC are familial = 4.5%				

Acrocentric chromosomes				

13	2	4	13	19
13/21	18	37	30	85
14	14	30	35	79
14/22	11	24	9	44
15	95	286	396	777
21	4	13	4	21
22	23	406	29	458
Acrocentric unclear origin	1	0	4	5
Summary	168	800	520	1488 *968 cases with known parental origin*

i.e. 168/968 of all reported detailed characterized acro sSMC are familial = 17.4%;				

Overall-Summary	202	1520	672	2394 *1520 cases with known parental origin*

i.e. 202/1520 of all reported detailed characterized sSMC are familial = 13.3%				

As mentioned above, acrocentric-derived sSMC are very likely to present as inverted duplicated derivatives. They are thought to arise during meiosis due to a U-type exchange of sister chromosomes. This usually leads to a partial tetrasomy of genetically irrelevant short arms, and hence these sSMC are more likely to be associated with normal phenotype than non-acrocentric derived sSMC [1].

**Figure 2:**
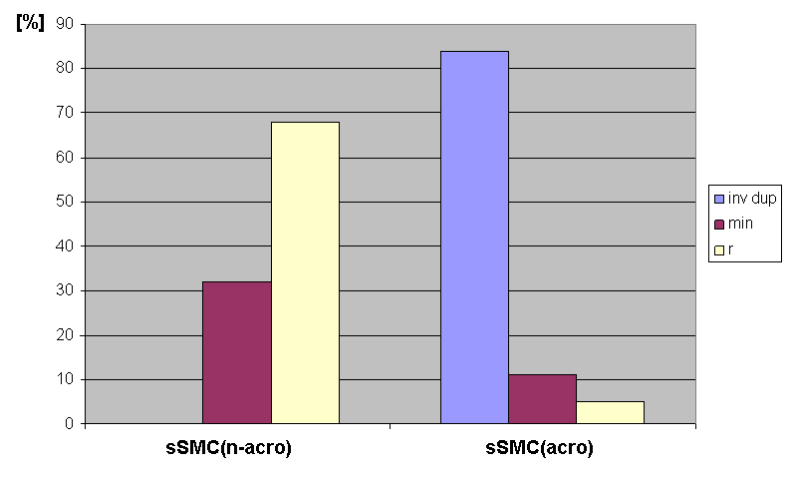
**The shape of familial sSMC is summarized according to the sSMC homepage.** In non-acrocentric chromosome-derived sSMC (sSMC(n-acro)) only ring (r) and minute (min) chromosomes are to be observed, in acrocentric chromosome derived sSMC (sSMC(acro)) predominantly inverted duplication (inv dup) followed by minute and ring chromosomes are present. The abbreviation 'min' is applied here according to the definition of 'minute chromosome' of Crolla (1998).

Isochromosome 8p, 9p, 12p and 18p syndromes are examples of non-acrocentric sSMC which can arise by a similar U-type exchange mechanism; such derivative chromosomes are very unlikely to become 'familial markers' due to the associated severe clinical phenotype. As shown in Fig. 2, the distribution of shapes of familial sSMC differs according to their origin: in sSMC(acro) the inverted duplicated derivatives are predominant, followed by minutes and rings; in sSMC(n-acro) only ring and minute shaped sSMC can be found. Potentially familial non-acrocentric derived sSMC are those that lead to clinically irrelevant genetic imbalances. Such regions are known throughout the whole human genome [Barber, 2005], but especially for at least 17 of the 46 centromere-near regions of the human chromosomes [6].

Recently, it was recognized that there is an as yet unexplained doubled transmittance-rate of sSMC via the maternal compared to the paternal line [3,4]. i.e. there is a natural selection against (*de novo*) sSMC mainly during spermatogenesis. If sSMC(acro) really would tend to develop to something like 'human B-chromosomes' they should be more stable in their inheritance throughout the generations than sSMC(n-acro). And according to [4] such a tendency really is observable: sSMC(n-acro) are transmitted 3.8-fold less frequently via paternal than maternal line, while in sSMC(acro) this rate is only 2.1-fold diminished. Thus, there could exist a subset of familial sSMC(acro) already behaving in a similar way to B-chromosomes and hence beginning to spread in the population.

It was proposed that the morphology of most mammalian chromosomes is determined by non random segregation during female meiosis [22]. The direction of nonrandom segregation may be variable in different species and it depends on the polarity of the meiotic spindle, which determines if the partner with the greater number of centromeres will go to the oocyte or to the polar body. According to this theory, humans are species with typical female meiosis bias towards fewer centromeres, i.e. karyotype evolution should move towards reduction of chromosomes and accumulation of metacentrics (resulting from Robertsonian fusions). Actually, the last macro-rearrangement in human karyotype evolution is a fusion of two ancestral acrocentrics resulting in submetacentric human chromosome 2 further supports this idea. B chromosomes should occur much more often in animals with centromeric drive favoring more centromeres, i.e. with acrocentric karyotypes, like known in rodents [23]. This would imply that in humans, appearance and maintenance of additional chromosomes would be against this bias. Only very strong positive selection or changes in centromeric drive mechanisms may favor fixation of B chromosomes in human populations. On the other hand there are some exceptional mammalian species where B-chromosomes occur in genomes with low number of metacentric chromosomes. In the red fox (*Vulpes vulpes*, 2n = 34+Bs) there are from 1 to 8 typical B-chromosomes in most animals studied, although it's karyotype evolution was accompanied by Robertsonian and non-Robertsonian fusions of ancestral canid chromosomes [24]. Discovery of genes on fox B-chromosomes [25] may reflect positive selection that contributed to fixation of those elements in fox populations.

## Conclusion

Inv dup(15) or inv dup(acro) chromosomes fit to the following prerequisites of B-chromosome behavior: (1) relatively high transmission rate, (2) recurrent origin (3) predominantly neutral on fitness – as far as yet known, (4) on the way to a polymorphic status in population. However, they did not yet acquire (5) some differences in molecular nature in respect to A chromosomes. The latter is the main condition of the two sSMC cases stainable only by DNA derived from themselves.

In summary, it is established that sSMC are a heterogeneous and special group of human derivative chromosomes, and are associated with abnormal phenotype in approximately 30% of cases [1,3,6,10]. Presently, there is no evidence to suggest that they are, in general, analogous to B chromosomes [13]. However, as outlined here, some sSMC subgroups do show B chromosome-like characteristics, and further studies may identify more such characteristics. At present one suggest that some sSMC can develop, or already have started to developed towards B chromosomes.

## Methods

### Cytogenetics and molecular cytogenetics

Banding cytogenetics (GTG-banding and NOR-staining) was done on metaphase cells derived from peripheral blood of the new patient with an sSMC only stainable by itself.

The sSMC was characterized by microdissection and reverse painting [7].

### Review of the literature

The sSMC-related literature is collected from [10].

## Competing interests

The authors declare that they have no competing interests.

## Authors' contributions

NK performed glass needle based chromosome microdissection and KM, VT JV, and NK did corresponding molecular cytogenetic studies. CM detected the patient with the first sSMC stainable only by DNA derived from itself and provided an EBV cell line. NR detected the patient with the first sSMC stainable only by DNA derived from itself and provided microdissection derived DNA of that patient. TL, VT, JV and KM have been involved in drafting the manuscript and revising it critically for important intellectual content. All authors read and approved the final manuscript.
